# Non-starch polysaccharide-degrading enzymes improve intestinal health by reducing digesta viscosity and gut permeability in young broilers fed high-fiber diets

**DOI:** 10.1016/j.vas.2026.100753

**Published:** 2026-06-27

**Authors:** Ester Vinyeta, Yueming Dersjant-Li, Abiodun Bello, Kirsty Gibbs, Susan Arent, Regiane R. Santos

**Affiliations:** a IFF Animal Nutrition & Health, 2342 BH Oegstgeest, The Netherlands; b IFF Animal Nutrition & Health, Edwin Rahrs Vej 38, 8220 Brabrand, Denmark; c Schothorst Feed Research B.V., PO Box 533, 8200 AM Lelystad, The Netherlands

**Keywords:** Beta-glucanase, Broiler, Gut permeability, Intestinal health, Viscosity, Xylanase

## Abstract

Young broilers are sensitive to dietary stressors such as increased level of non-starch polysaccharide (NSP) because their intestinal morphology and function are still developing and not yet fully mature. Viscous grains such as rye and barley with high NSP content can increase digesta viscosity, impair nutrient absorption, increase gut leakage and reduce performance. This study evaluated the effect of a xylanase-β-glucanase enzymes combination (XB) and a consensus bacterial 6-phytase variant (PhyG) on gut health and growth performance in young broilers (0 to 14 d old) fed diets containing rye and barley. A randomized complete block design was used and 180 Ross 308 male chicks was assigned to 5 treatment diets with 6 replications per treatment: 1) corn-wheat-soybean meal-based positive control (PC); 2) the PC reformulated with addition of rye and barley served as negative control (NC); 3) the NC supplemented with 1,000 phytase units (FTU)/kg of phytase (NC+PhyG); 4) the NC supplemented with phytase and XB (at 100g/MT providing 1,220 XU/kg xylanase and 152 U/kg β-glucanase, NC+PhyG+XB100), and; 5) the NC supplemented with the same phytase dose but XB at double dose (NC+PhyG+XB200). All diets were fed ad libitum as a mash. Compared with the PC, the NC increased (*P* < 0.05) ileal and jejunal digesta viscosity, increased intestinal permeability and reduced overall weight gain, all of which were fully ameliorated (*P* > 0.05) by the NC+PhyG+XB200 treatment. The results indicate that combining phytase and XB is efficacious for supporting gut health and maintaining growth performance in young broilers from 0-14 d of age fed a NSP-rich diet.

## Highlights

• NSP-rich diets increased viscosity, gut permeability and reduced growth.
• Enzyme blend (phytase + xylanase/β-glucanase) improved gut function.
• Enzymes reduced viscosity and enhanced performance in NSP diets.
• Enzyme response was dose-dependent, with greater effects at high dose.
• High enzyme dose restored gut health parameters and weight gain.

## 1 Introduction

Maintaining an effective gut barrier during the first two weeks of life is critical for optimizing nutrition and subsequent growth in modern broiler breeds (Wang et al., 2025). It has been reported that gut morphology is not maximized until 10 d of age (Sklan, 2001) and the immune system does not fully mature until 30 to 34 d of age in cage-reared broilers (Song et al., 2021). Hence, young birds are particularly vulnerable to stressors that can compromise gut health and impact on growth performance. Stressors may include pathogen challenge, heat stress, mycotoxins, feed restriction and inclusion of feed raw materials rich in non-starch polysaccharides (NSP) such as barley and rye (Santos et al., 2019; Santos et al., 2021a, 2021b; Yan et al., 2021). However, there are still knowledge gaps and more research is needed to evaluate the nutritional solutions that can reduce stress and improve gut health during this age.

The major source of energy in broiler nutrition is corn (Syamsu et al., 2025), on the cost and sustainability ground, more alternative ingredients are being used. Barley and rye are readily available in Europe and increasingly being used as an alternative source of energy in the diet for broilers. Demand of these alternative ingredients is expected to increase further over the next few years (USDA, 2024). These cereals have higher fiber and associated NSP content than corn. The NSP content (DM basis) in corn is 81 g/kg (1% soluble, 99% insoluble), it is higher in barley 167 g/kg (27% soluble, 73% insoluble) (Perera et al., 2022) and rye, 155 g/kg (35% soluble; 65% insoluble) (Arczewska-Wlosek et al., 2019). The high NSP content can be both beneficial and deleterious for nutrient utilization and growth. Insoluble fiber may improve starch digestion, gut motility and bird behavior (Hetland et al., 2019). However, soluble NSP can form a gel in the gastrointestinal tract (GIT) which increases digesta viscosity (Nguyen et al., 2021a), leading to increases in retention time and altering gut microflora. This may also result in dysbiosis (Choct et al., 1996; Nguyen et al., 2021a) and affect gut morphology impairing nutrient absorption (Langhout et al., 2000). The immature gut of young broilers is less able to move a viscous digesta along the GIT, due to reduced contractile activity (Smulikowska et al., 2002). Ultimately, in the absence of supplemental enzymes, barley and rye inclusion can impair growth performance (Friesen et al., 1992; Marquardt et al., 1994; van Krimpen et al., 2017).

Phytase and NSP-degrading enzymes are widely used to improve nutrient availability and utilization in broiler production (Selle et al., 2023; Mohamed et al., 2024). Phytase has proven efficacy for increasing the digestibility of phosphorus from plant-derived phytate and can also improve the digestibility of other minerals, amino acids, starch and energy, leading to improved growth performance in broilers (Dersjant-Li et al., 2022; Sobotik et al., 2023). Recent studies have indicated a beneficial effect of phytase on intestinal integrity and taxonomic composition of the intestinal microbiome in broilers (Moita et al., 2021; Shi et al., 2024), which may be linked to reduced phytate abundance but this is not fully understood and is likely to be multifactorial. Xylanase and β-glucanase have been shown to be effective in a range of diet types for reducing the antinutritive effects of NSP by degrading fiber, reducing digesta viscosity, stimulating beneficial bacteria in the hindgut and improving growth performance (Gilani et al., 2021; Morgan et al., 2021; Munyaka et al., 2016). However, their effects on indicators of intestinal health in broilers fed NSP-rich diets, such as intestinal epithelial permeability and morphology, have not received much research attention. Improved understanding of the mechanism of effect of these enzymes on gut health when used in NSP-rich diets is needed.

It has been reported that inclusion of 10% rye in a diet not supplemented with enzymes causes intestinal stress and symptoms of mild (sub-clinical) challenge in young birds (Santos et al., 2021a). This is characterized by increased epithelial permeability, reduced villus height (VH)-to-crypt depth (CD) ratio, increased cell damage in the jejunum at 14 d of age, and reduced expression of epithelial tight junction proteins at 28 d of age (Santos et al., 2021a). In this study, a similar condition of sub-clinical challenge in young broilers (0 to 14 d of age) was created by feeding a mixed-cereal diet containing 20% fibrous grains (5% barley and 15% rye), rich in NSP. The hypothesis is that supplementation of a phytase and a xylanase and β-glucanase combination to this diet would improve intestinal health and support growth performance responses.

## 2 Materials and methods

The experiment was carried out in accordance with European Directive 2010/63/EU and the regulations in force in the Netherlands for the care and use of animals in research. In addition, the animal procedures and experimental protocols were evaluated and approved by the Ethical Committee on Animal Experiments (Ethische Toetsing Dierproeven) of Schothorst Feed Research B.V. prior to the commencement of research.

### 2.1 Birds and housing

A total of 180 Ross 308 newly hatched male broiler chicks were obtained from a commercial hatchery and randomly assigned to 30 floor pens with six birds per pen. Each pen had a surface area of 2.2 m^2^ (2.2 × 1 m) with one feeder and three drinking nipples). Pens contained wood shavings as bedding material and were located in an environmentally controlled animal house. The temperature in the animal house was maintained initially at 34 °C and gradually decreased from the second day onwards to reach 27 °C at 14 d of age. The lighting regime was light-dark (LD) 24:0 h during d 1, LD 22:2 h during d 2 and changed to 10 (L): 4 (D): 8 (L): 2 (D) h thereafter, in compliance with EU legislation that prescribes a maximum of 6 h of darkness and at least one period of 4 h of uninterrupted darkness. Access to diets and water was ad libitum.

### 2.2 Experimental design and diets

The experiment was carried out as a randomized complete block design. Pen location in the animal house was the blocking factor and randomization was performed at chick placement. There were five experimental treatments and six replicate floor pens per treatment.

Treatments comprised of: 1) a commercially representative corn-wheat-SBM-based positive control diet (PC) low in soluble NSP (3.42 %), containing 2,850 kcal/kg AME_n_, 0.93 % Ca, 0.38 % retainable P (total P intake – total P excretion) and 1.2 % digestible Lys; 2) a negative control (NC), high in soluble NSP (4.02 %), containing 15 % rye and 5 % barley (as a replacement for corn) and reduced in Ca and retainable P (by 0.2 % and 0.17 % points, respectively, vs. PC, based on the expected contribution of phytase); 3) the NC supplemented with a consensus bacterial 6-phytase variant (PhyG) at 1,000 phytase units (FTU)/kg (NC+PhyG); 4) the NC+PhyG supplemented with a xylanase–β-glucanase combination (XB) to provide xylanase at 1,220 xylanase units (**XU**)/kg and β-glucanase at 152 units (U)/kg (NC+PhyG+XB100), and; 5) the NC+PhyG supplemented with XB to provide 2,440 XU/kg xylanase and 304 U/kg β-glucanase (NC+PhyG+XB200). The soluble NSP level of 3.42–4.02% was selected to reflect practical dietary ranges, within which even small variations are known to significantly influence digesta viscosity and nutrient utilization in broilers (Nguyen et al., 2021a; Nguyen et al., 2022). Oat hulls were added to the NC diet as a filler ingredient. The full ingredient and calculated nutrient composition of the basal diets is given in Table 1. The phytase was produced in *T. reesei.* The xylanase–β-glucanase combination was a co-granule comprising an endo-1.4-β-xylanase (EC 3.2.1.8) produced in *T. reesei* and an endo-1,3(4)-β-glucanase (EC 3.2.1.6) produced in *T. reesei*. All enzymes were supplied by Danisco Animal Health & Nutrition (IFF), Oegstgeest, The Netherlands. The NC diet was prepared as a single batch and subsequently split into four sub-batches to which the enzymes were added according to treatment. The final diets were mixed thoroughly to ensure a homogeneous distribution of ingredients and enzymes. All diets were fed in mash form.

**Table 1:** Ingredient and calculated nutrient composition of the basal diets.

	PC	NC
Ingredients, % (as-fed basis)		
Soybean meal	31.44	28.43
Wheat	20.00	20.00
Corn	35.73	13.72
Rye	-	15.00
Barley	-	5.00
Rapeseed meal	5.00	5.00
Corn gluten meal	0.00	1.78
Oat hulls	0.00	1.02
Palm oil	0.00	3.25
Soybean oil	3.65	3.49
Monocalcium phosphate	1.18	0.30
Salt	0.22	0.19
Limestone	1.58	1.45
Sodium bicarbonate	0.16	0.20
L- lysine HCl	0.22	0.30
DL-methionine	0.27	0.28
Threonine	0.05	0.08
Arginine	0.00	0.03
Valine	0.00	0.01
Vitamin & mineral premix^1^	0.50	0.50
Chemical composition, g/kg (unless otherwise stated)		
AMEn, kcal/kg	2,850	2,854
DM	882	887
Ash	61.44	51.48
CP	223	223
Crude fat	61.10	88.50
CF	26.22	30.13
Starch	345	314
Ca	9.33	7.33
Total P	6.55	4.50
Retainable P	3.79	2.15
Phytate-P	2.40	2.26
Na	1.40	1.40
SID Lys	11.90	11.91
SID Met	5.68	5.73
SID Cys	2.85	2.82
SID Met+Cys	8.53	8.54
SID Thr	7.26	7.27
SID Trp	2.29	2.19
Total NSP	15.53	16.72
Soluble NSP	3.42	4.02
Insoluble NSP	12.11	12.70

1 Supplied per kilogram of diet: vitamin A (retinyl acetate), 10,000 IU; vitamin D3, 3,333 IU; vitamin E (dl-α-tocopherol), 50 IU; vitamin K3 (menadione), 2.5 mg; vitamin B1 (thiamine mononitrate) 2.5 mg; riboflavin, 7.5 mg; D-pantothenic acid, 15 mg; vitamin B6, 5.0 mg; vitamin B12, 0.025 mg; niacin, 50 mg; folic acid, 1.5 mg; biotin, 0.25 mg; Fe: 50 mg/kg; I, 2.0 mg; Cu: 12.5 mg/kg; Mn: 75 mg/kg; Zn: 70 mg/kg; Se: 0.25 mg/kg. NSP, non-starch polysaccharides.

### 2.3 Sampling, measurements and chemical analysis

Growth performance: Birds were checked daily for health and mortality and any dead birds were removed and weighed. Birds were weighed on a per pen basis on each of d 0, 7 and 14. Body weight gain (BWG) was calculated per week (0 to 7 and 7 to 14 d of age). Feed intake (FI) was determined per pen based on the difference between the weight of feed provided and that of any residual feed at the end of each week. Feed conversion ratios (FCR) were calculated per week with correction for mortality.

Intestinal permeability: At 14 d of age, four birds per pen received 2.2 mg/ml (1 ml per bird) of Fluorescein isothiocyanate-dextran (FITC-d; Sigma Aldrich Co., St. Louis, MO, USA) via oral gavage, for the estimation of intestinal paracellular permeability, and euthanized 150 min later for tissue and blood collection, based on the method as previously described (Tellez et al., 2014). Birds were euthanized by CO_2_ gasification followed by exsanguination and blood was collected via jugular vein puncture. Blood was centrifuged at 150 × g for 15 min to obtain serum which was stored in darkness at −20 °C until analysis. The FITC-d levels in serum were measured as described by Baxter et al. (2017), using an Infinite 200 Pro plate reader (Tecan, Männedorf, Switzerland).

Viscosity: Digesta was collected from the jejunum and ileum of the same four birds that were euthanized following FITC-d administration. Samples were pooled per pen, and per intestinal section. Viscosity was determined as described by Tellez et al. (2014) with minor modifications. In brief, samples were centrifuged at 3,500 × g at 4 °C for 10 min, the supernatant was filtered and kept on ice. Viscosity was determined using a DV-II LVCP Brookfield digital cone-plate viscometer (Brookfield Engineering, Middleboro, MA, USA) with the viscosimeter cup maintained at 20 °C during measurement. Viscosity was expressed in centipoise (cP) units.

Intestinal morphology and morphometry: Samples of jejunal and ileal tissue were collected from an additional two birds per pen at 14 d of age, euthanized as before, and fixed in buffered formalin for histological staining and morphological analysis. Histological sections were stained in haematoxylin-eosin, scanned using a NanoZoomer-XR (Hamamatsu Photonics KK, Hamamatsu, Japan), viewed and analyzed through the manufacturer’s software (NDP.view2 and NDP.analyze, respectively). Villus height, CD and villus area (VA) were measured for each individual bird (10 villi per intestinal segment) and an average taken, as previously described by Santos et al. (2014).

Diets: The PC and NC diets were sampled for nutrient analysis and all diets were sampled for enzyme analysis (β-glucanase was not analyzed because it was added together with xylanase as a co-granule, and therefore, the measured activity of xylanase was used as a proxy to confirm the β-glucanase activity). Diets were analyzed for moisture (NEN-ISO, 1999a), ash (NEN-ISO, 2003), crude protein (CP, NEN-EN-ISO, 2008), crude fiber (CF, NEN-EN-ISO, 2001), crude fat (NEN-ISO, 1999b) and starch (NEN-EN-ISO, 2005) at Schothorst Feed Research. The Ca and P contents were analyzed by Eurofins (Denmark) and determined by inductively coupled plasma optical emission spectroscopy (ICP-OES) in accordance with Commission Regulation (EC) 152/2009 (European Commission, 2009). Phytate concentrations were determined at Danisco Animal Nutrition Research Centre (IFF, Brabrand, Denmark) using the HPLC method described by Christensen et al. (2020), modified from that of Skoglund et al. (1998). The in-feed enzyme activities were analyzed also by Danisco Animal Nutrition Research Centre. Phytase activity was analyzed according to a modified version of AOAC method 2000.12 (Engelen et al., 2001), where one FTU was defined as the quantity of enzyme that released 1 µmol of inorganic phosphate from a 0.0051 mol/L sodium phytate substrate per minute at pH 5.5 at 37 °C. Xylanase analysis was conducted in duplicate and reported as activity units as described by Romero et al. (2013). One XU was defined as the amount of enzyme that released 0.48 μmol of the reducing sugar xylose from wheat arabinoxylan per min at pH 4.2 and 50 °C.

### 2.4 Statistical analysis

Data were analyzed by one-way ANOVA including treatment as a fixed effect and block as a random effect. Pen was the experimental unit. Means separation was performed using Tukey’s HSD test. All statistical analyses were performed in JMP (version 16.0; SAS Institute Inc., Cary, NC, USA; JMP, 2021). A *P* value of ≤ 0.05 was considered statistically significant, whereas 0.1 > *P* > 0.05 was considered a statistical tendency. In addition, linear response was analyzed for increasing XB dose including treatment NC+PhyG, NC+PhyG+XB100, NC+PhyG+XB200, as these diets all contained phytase and with increasing XB dose levels (0, 100 and 200 g/MT). The linear response was used because the limited data points (only 3 dose levels).

## 3 Results

### 3.1 Diet analysis

Analyzed nutrients and enzyme activities in the diets are presented in Table 2. The analyzed CP is lower in all NC diets than formulated. Phytase activities, after subtraction of the phytase activity in the NC, the values were consistently close to the target of 1,000 FTU/kg. Xylanase activities in the NC+PhyG+XB100 and NC+PhyG+XB200 diets were considered adequate to verify enzyme inclusion; the intended spacing between the XB100 and XB200 dose levels was achieved.

**Table 2:** Analyzed nutrients (g/kg, as is) and enzymes in the treatment diets.

	PC	NC	NC+PhyG	NC+PhyG+XB100	NC+PhyG+XB200
Nutrients					
DM	884	888	889	889	890
Ash	58	45	46	48	48
CP	230	189	198	202	204
Crude fat	63	85	85	90	90
CF	28	33	33	33	34
Starch	342	368	368	353	351
Ca	9.82	7.75	-	-	-
P	6.80	4.21	-	-	-
Phytate-P^1^	2.51	2.28			
Enzyme activities					
Phytase, FTU/kg	290	637	1,982	1,887	1,801
Xylanase, U/kg	43	36	48	1,598	2,961

1 Ca, P and phytate-P were only analyzed in the control diets (PC and NC).

### 3.2 Digesta viscosity

**Fig. 1:**
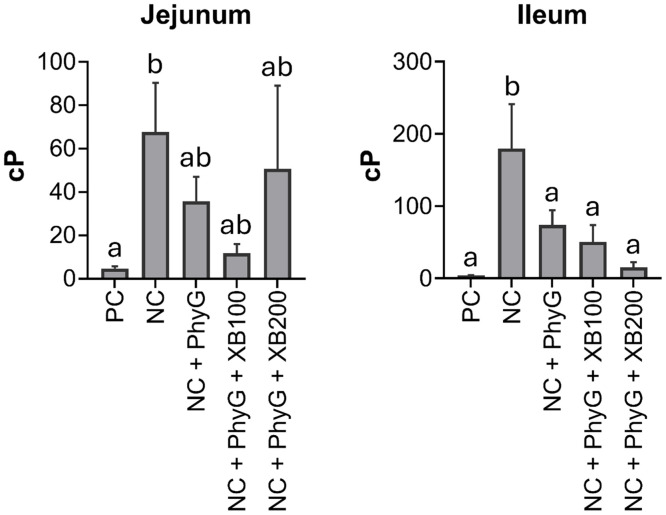
Mean (± SEM) viscosity (centipoise units, cP) in the jejunum and ileum of broilers, by treatment, at 14 d of age. ^a,b^Different lower case letters indicate a significant difference at *P* < 0.05. For XB dose response analysis, *P* =0.68 for jejunum and *P* = 0.052 for ileal digesta viscosity. The experiment was carried out with 6 repetitions, where each repetition represents the pooled intestinal content from 4 broiler chickens per pen.

The added barley (5%) and rye (15%) in the NC resulted in a substantial increase in jejunal and ileal digesta viscosity relative to the wheat- and corn-based PC (+64 and +182 cP, respectively, at 14 d of age; *P* < 0.05), as shown in Fig. 1. The major effect of the addition of the phytase and XB enzymes, regardless of XB dose level, was a reduction (*P <* 0.05) in ileal digesta viscosity relative to the NC, to levels that were similar (*P* > 0.05) to the PC in all three enzyme-supplemented treatments (57.1, 50.5 and 18.9 cP in NC+PhyG, NC+PhyG+XB100 and NC+PhyG+XB200, respectively, vs. 4.0 cP in the PC; Fig. 1). A tendency for a linear decrease in ileal digesta viscosity (*P* = 0.052) was observed as XB dose increased. The enzyme supplementation showed intermediate response on jejunal viscosity between PC and NC.

### 3.3 Intestinal morphology

**Fig. 2:**
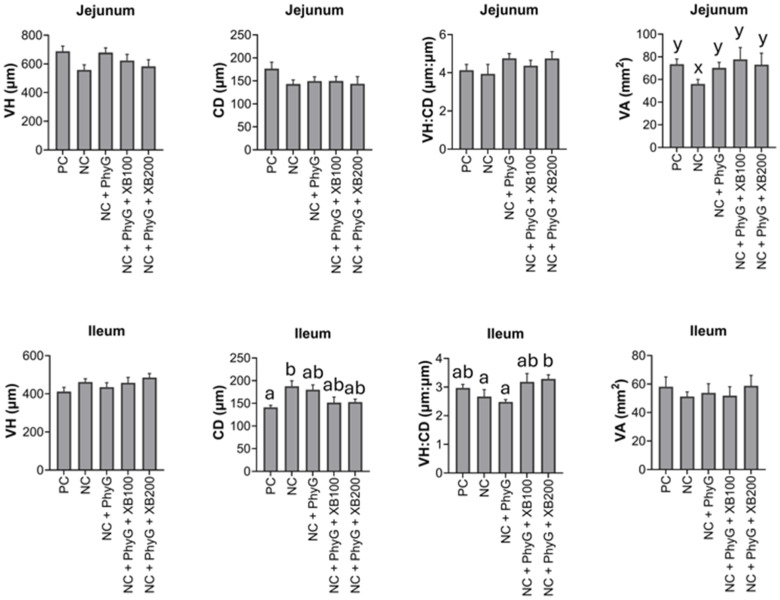
Mean (± SEM) villus height (VH: μm), crypt depth (CD: μm), villus height:crypt depth ratio (VH:CD; μm;μm) and villus area (VA; mm^2^) of the jejunum and ileum of broiler chickens fed the experimental diets, at 14 d of age. ^a,b^Different lower case letters indicate a significant difference, at *P* < 0.05 and ^x, y^ indicate a tendency (*P* < 0.1). For XB dose response analysis, *P* (linear) = 0.001 for ileal VH:CD. The experiment was carried out with 6 repetitions, where each repetition represents intestinal tissues from 2 chickens per pen.

**Fig. 3:**
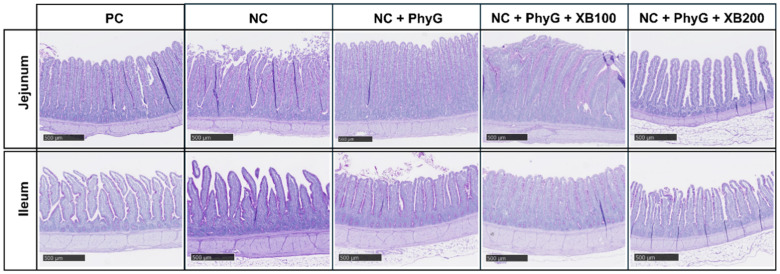
Illustrative images of PAS-haematoxylin-stained sections of the jejunum and ileum of broilers fed the experimental diets. Scale bars: 500 μm.

The effect of treatment on intestinal morphological measurements is shown in Fig. 2 while representative microscopic images of stained jejunal and ileum sections from broilers in each treatment are shown in Fig. 3. Treatment had a significant effect on ileal CD (*P* < 0.05) and VH:CD ratio (*P* = 0.01) and tended to affect jejunal VA (*P* = 0.09). Fig. 3 indicates that in the ileum, the NC birds had generally shorter, less densely packed villi with a less uniform shape than the PC. The effect of the NC relative to the PC was that ileal CD was increased (*P* < 0.05) although VH:CD was not affected.

The NC+PhyG, NC+PhyG+XB100 and NC+PhyG+XB200 treatments numerically reduced ileal CD vs. NC but also not differ from PC (*P* > 0.05). The NC+PhyG+XB200 treatment increased (*P <* 0.05) ileal VH:CD compared with the NC or NC+PhyG, and to a level that is comparable to PC. Increasing XB dose linearly increased ileal VH:CD (*P* < 0.01).

### 3.4 Intestinal permeability

**Fig. 4:**
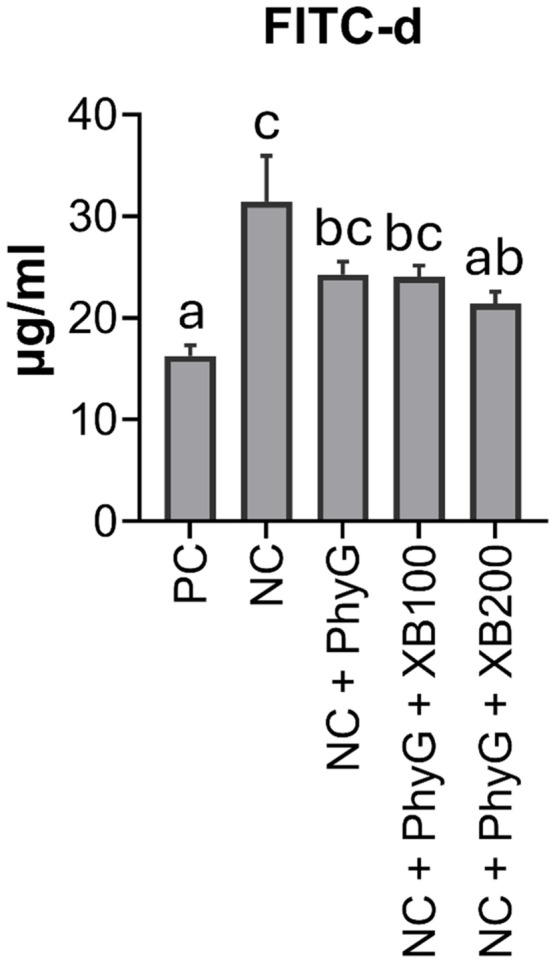
Mean (± SEM) FITC-d levels (μg/ml) in the serum of broilers fed the experimental diets, at 14 d of age. ^a-c^Different lowercase letters indicate a significant difference at *P* < 0.05, For XB dose response analysis, *P* (linear) = 0.093. FITC-d, fluorescein isothiocyanate-dextran. The experiment was carried out with 6 repetitions, where each repetition represents pooled intestinal content from 4 broiler chickens per pen.

Treatment had a significant effect on serum FITC-d levels at 14 d of age (*P* < 0.001, Fig. 4). The FITC-d level was higher in the NC than the PC (30 vs. 17.0 µg/ml), *P* < 0.05), reduced (*P* < 0.05) vs. NC in NC+PhyG+XB200, to a level not different from the PC. The NC+PhyG and NC+PhyG+XB100 treatments showed intermediate response between NC and PC (*P* > 0.05). A tendency (*P* = 0.092) for linear decrease on serum FITC-d levels was observed as XB dose increased.

### 3.5 Growth performance

Growth performance during the experimental period (0 to 14 d of age) is presented in Table 3. There was no mortality and FCR was unaffected by treatment.

**Table 3:** Mean BW (g), BW gain (BWG, g), feed intake (FI, g), and feed conversion ratio (FCR, g:g) of broilers fed the experimental diets from 0 to 14 d of age^1^.

	PC	NC	NC+PhyG	NC+PhyG+XB100	NC+PhyG+XB200	SEM	*P*-value	*XB dose P linear*^2^
Day 0, BW(g)	43.1	43.2	43.1	43.3	43.3	0.19	0.70	
*During 0 to 7 d of age*								
BW on d 7, g	145^b^	129^a^	129^a^	133^ab^	141^ab^	0.02	<0.001	0.02
BWG, g	102^b^	86^a^	86^a^	89^ab^	101^b^	3.20	<0.01	<0.01
FI, g	147^ab^	137^ab^	143^ab^	136^a^	158^b^	4.83	0.04	0.18
FCR, g/g	1.440	1.611	1.660	1.536	1.557	0.055	0.10	0.30
*During 7 to 14 d of age*								
BW on d 14, g	367^c^	284^a^	308^b^	323^b^	358^c^	5.55	<0.001	<0.001
BWG, g	222^c^	155^a^	179^b^	191^b^	213^c^	4.92	<0.001	<0.001
FI, g	345^ab^	280^a^	283^ab^	355^b^	321^ab^	21.1	0.03	0.23
FCR, g/g	1.563	1.696	1.596	1.861	1.502	0.126	0.33	0.75
*During 0 to 14 d of age*								
BWG, g	324^c^	241^a^	265^b^	280^b^	313^c^	5.52	<0.001	<0.001
FI, g	492^b^	398^a^	425^ab^	491^b^	477^b^	23.6	0.04	0.13
FCR, g/g	1.522	1.667	1.612	1.757	1.517	0.089	0.32	0.59

a-z Means in the same row without a common superscript are significantly different at *P* < 0.05.

1 The experiment was carried out with six repetitions, where each repetition represents one pen with six broiler chickens.

2 Linear response was analyzed for increasing XB dose including treatment NC+PhyG, NC+PhyG+XB100, NC+PhyG+XB200

During each week (0 to 7, 7 to 14) and cumulatively (0 to 14 d of age), BW and BWG were reduced (*P* < 0.05) in the NC compared with the PC (BWG reduced by 16 g/bird, 67 g/bird and 83 g/bird, respectively, vs. PC). In addition, for the overall period (0 to 14 d of age) FI was reduced (*P <* 0.05) in the NC relative to the PC (by 94 g/bird).

Phytase addition to the NC increased (*P <* 0.05) BWG during 7 to 14 d of age and overall (+24 g/bird and 24 g/bird, respectively). The addition of XB on top of the phytase to the NC increased BWG, with a linear increase with increasing XB dose for each week and overall (*P* < 0.05). During 0 to 7 d of age, BWG was greater (*P* < 0.05) in NC+PhyG+XB200 than NC+PhyG or NC, whereas during 7 to 14 d of age and overall, BWG was greater (*P* < 0.05) in both NC+PhyG+XB100 and NC+PhyG+XB200 than the NC, and greater (*P* < 0.05) in NC+PhyG+XB200 than NC+PhyG+XB100. For the periods 7 to 14 and 0 to 14 d of age, BWG was comparable in NC+PhyG+XB200 to PC (313 g/bird in NC+PhyG+XB200 and 324 g/bird in PC for the overall period; *P* > 0.05). Feed intake was comparable in both XB treatments to PC, for the overall period.

## 4 Discussion

### 4.1 Diet analysis

The formulated crude protein (CP) content was 22.3% for both PC and NC diets; however, analyzed CP was 23.0% in PC and averaged 19.8% in NC (single NC batch). The lower-than-expected CP in NC likely reflects that the actual CP content was lower in rye and barley than formulated values. This could have led to a lower actual digestible AA content than formulated in NC. The substantial difference in analyzed CP between PC and NC may have partially contributed to the observed performance differences, in addition to variations in NSP levels. Although this reduction in CP is unlikely to have confounded the evaluation of enzyme effects, the magnitude of the response may have been underestimated under conditions of increased digestible amino acid deficiency (Cowieson and Ravindran, 2008).

The analysed phytase activity was higher in NC than in PC, likely reflecting a greater contribution of intrinsic phytase from rye and barley. After correction for intrinsic phytase levels in the NC diet, the phytase activities were 1345, 1250, and 1164 FTU/kg for NC+PhyG, NC+PhyG+XB100, and NC+PhyG+XB200, respectively, confirming that the targeted inclusion level of 1000 FTU/kg was achieved. Analysed xylanase activity was 20–28% above target after subtraction of xylanase activity in basal diet, this is likely due to product overage.

### 4.2 Digesta viscosity

The tendency (*P* = 0.052) of linear decrease in ileal digesta viscosity with increase XB concentration suggests a dose-response effect of the XB enzymes. Kim et al. (2025) reported that supplementation with xylanase, either alone or in combination with β‑glucanase, significantly reduced ileal digesta viscosity in broiler chickens. However, information on the dose–response effects of XB on ileal digesta viscosity remains limited. Gorenz et al. (2022) previously reported a linear dose-response relationship between xylanase supplementation and ileal digesta viscosity in broilers fed a wheat-based diet. Several previous studies on xylanase supplementation have reported reduced ileal digesta viscosity when the enzyme was added to viscous diets based on wheat, rye or barley (Arczewska-Wlosek et al., 2019; Barasch and Grimes, 2021; Mathlouthi et al., 2002) in birds from 0 to 21 d of age or older, but none has reported data specifically for the 0 to 14 d. Anwar et al. (2023a, 2023b, 2023c) reported that supplementation of xylanase alone or in combination with phytase consistently reduced digesta viscosity in broilers at around 35 days of age. The mode of action of XB on viscosity may be explained by the proven hydrolyzing activity of xylanase and β-glucanase against soluble NSP that are contained within the hemicellulose fraction of the cell walls, principally xylans in rye and barley (Perera et al., 2022) and β-glucans in barley (De Arcangelis et al., 2019). This activity would have reduced the abundance of soluble NSP in the digesta and thereby reduced digesta viscosity. Fewer studies linking phytase with viscosity are available and the mechanism for its beneficial effect in treatment NC+PhyG relative to the NC is less clear. The effect of phytase on digesta viscosity is likely mediated through disruption of phytate-associated complexes rather than direct NSP hydrolysis. Accordingly, reductions in viscosity with phytase supplementation have been reported, although the magnitude of effect is smaller than with NSP-degrading enzymes. Anwar et al. (2023a) indicated that xylanase is the primary driver of viscosity reduction in wheat-based diets, whereas phytase contributes indirectly, with combined supplementation improving overall nutrient utilization and performance.

The results of the present study indicate that enzyme supplementation exerted a modest effect on jejunal digesta viscosity but a more pronounced reduction in the ileum. The greater variability observed in jejunal viscosity likely reflects the dynamic nature of NSP solubilization and enzymatic hydrolysis in the proximal small intestine, where rapid changes in digesta composition and enzyme–substrate interactions can amplify between-bird variation (Choct et al., 1999; Nguyen et al., 2021a). In contrast, the more consistent response in the ileum suggests cumulative NSP degradation along the intestinal tract, resulting in a clearer viscosity-lowering effect at the distal site (Bedford & Schulze, 1998; Choct et al., 1999). In addition, baseline viscosity is typically higher in the ileum, which may increase the sensitivity for detecting enzyme effects at this site (Choct, 2006; Van Hoeck et al., 2021).

### 4.3 Intestinal morphology

In the present study, it was observed that birds fed NC diet had shorter, less densely packed villi, this is similar to that observed by Wu et al. (2004) in broilers fed a highly viscous wheat-based diet (67% wheat). Tierlynck et al. (2009) also observed impaired intestinal morphology in birds fed a wheat-rye-based diet (50% wheat, 5% rye) compared with a corn-based diet, including increased villus fusion and a thinner tunica muscularis. Increased CD is indicative of an inflammatory process involving increased cell proliferation in the crypts as a response to damage (Ducatelle et al., 2018; Rysman et al., 2023) and indicates mild intestinal challenge induced by the rye- and barley-based diet. Increased CD has been reported to correlate negatively with production outcomes under field conditions (Rysman et al., 2023).

The VH:CD ratio is an established biomarker of intestinal morphology and absorptive capacity, and is positively associated with nutrient absorption and growth performance (Nguyen et al., 2021b). In the present study, a linear increase in ileal VH:CD ratio was observed with increasing XB dose. Similarly, Kim et al. (2025) demonstrated that xylanase and β‑glucanase supplementation restored the VH:CD ratio in broilers fed high-NSP, energy-deficient diets. Data on the effect of β-glucanase specifically is still relatively limited, but several previous studies have noted beneficial changes in VH or CD measures in response to xylanase supplementation, with or without phytase. Other studies have reported that supplementation with phytase, xylanase, or their combination increased villus height (VH) and reduced crypt depth (CD) in specific segments of the small intestine (Wu et al., 2004; Wang et al., 2021, 2024). Ceylan et al. (2024) noted increased VA and total goblet cell number in xylanase-supplemented birds fed a barley-based diet. The results of the present study are partially consistent with these earlier findings and suggest that the XB in combination of phytase at the higher dose level ameliorated the negative effect of the NSP-rich diet on ileal morphology, with potential to benefit nutrient absorption.

### 4.4 Intestinal permeability

The results from the present study indicate that the NSP-rich NC diet increased intestinal permeability, as expected given previous reports of NSP-rich diets impair the integrity of the intestinal epithelial barrier by disrupting the functioning of tight junctions (Baxter et al., 2019; Chen et al., 2015; Santos et al., 2021a). Increased permeability leads to inflammation and compromised gut integrity which results in the leakage of substances across the gut barrier into the bloodstream (Santos et al., 2021a; Tellez et al., 2014). It was shown that diets rich in soluble-NSP stimulated lymphocyte infiltration in the gut wall as well as apoptosis of epithelial cells (Teirlynck et al., 2009), both of which may have contributed to the reduced epithelial barrier integrity of NC-fed birds in the present study.

The tendency for linear decrease in serum FITC-d levels with increasing XB dose and the comparison in the enzyme-supplemented treatments suggest that the higher XB dose level was effective in maintaining intestinal epithelial integrity in birds fed the NSP-rich rye- and barley-based diet, providing protection comparable to that observed in the PC and countering the negative effect of the NSP-rich diet. Limited information is available on this beneficial effect on epithelial permeability from supplemented xylanase (and β-glucanase). Daneshmand et al. (2023) reported that supplementation of a wheat-barley-rye-based diet with xylanase and glucanase did not significantly improve serum FITC-d levels in *Eimeria*-challenged broilers although it did increase the population of beneficial *Lactobacillus* in the cecum. Wang et al. (2021) reported reduced levels of pro-inflammatory cytokines in the ileum of broilers, and Liu et al. (2012) observed reduced apoptosis and increased expression of tight junction proteins in the ileum of broilers fed a wheat-based diet supplemented with xylanase. The improvement in epithelial permeability observed in the present study is consistent with the enhancements in intestinal morphometry and digesta viscosity, suggesting it may form part of the mechanism underlying the observed gains in growth performance that have been reported in numerous studies.

### 4.5 Growth performance

The performance results from the present study demonstrate the negative effect of the NSP-rich rye- and barley-based diet on growth performance in the absence of enzyme supplementation, and are consistent with the findings of earlier studies (Arczewska-Wlosek et al., 2019; Rawash et al., 2023; Tellez et al., 2014). The reduced BWG of NC birds may be related to the increased digesta viscosity since these two variables are known to be inversely correlated during 1 to 21 d of age (Ayres et al., 2019). Possible mechanisms for this effect include: 1) the direct binding of NSP to nutrients, especially lipids (Singh and Kim, 2021); 2) increased ileal fermentation by microorganisms (Choct et al., 1996); 3) reduced gut motility (Smulikowska et al., 2002), and, 4) reduced diffusion rate of digestive enzymes, feed substrates and their metabolic products (Jha and Mishra, 2021), all these can lead to reduced nutrient absorption.

The positive effect of phytase addition to the NC on growth performance may be related to the documented capacity of PhyG to release P and other nutrients (Ca, amino acids, starch, energy and Na) from feed raw materials via its degradation of phytate, which can have a direct or indirect beneficial effect on nutrient utilization for growth (Dersjant-Li et al., 2022; Selle et al., 2023).

The linear increase in BWG with XB dose indicates a dose‑dependent response, consistent with ileal viscosity. Although limited data are available on the dose-response effect of the XB, a quadratic effect of xylanase supplementation on broiler BWG was observed in a recent meta-analysis of 53 published studies (Inayah et al., 2022). The similarity in all growth performance responses between birds receiving the higher XB dose plus phytase and those fed the PC indicates that this XB dose, in combination with 1,000 FTU/kg phytase, fully compensated for the negative effects of the NSP-rich diet on performance in broilers from 0-14 d of age. This highlights the need to consider the optimal XB inclusion rate when formulating NSP-rich diets with xylanase and β-glucanase for young broilers, taking into account the inclusion level of rye, barley or other viscous grains.

As the study focused on the effects of enzymes on gut leakage in young broilers, it was terminated at day 14. Comparable responses have been reported in the literature. Vargas et al. (2024) demonstrated that, in a low-energy diet, a higher xylanase dose (4800 U/kg) was required to restore BW and FCR to the level of the positive control at day 14, whereas a lower dose (1200 U/kg) was sufficient to recover FCR at day 28. This suggests that higher enzyme inclusion may be more beneficial during early life stages.

In the present study, the NC+phyG+XB200 treatment improved FI and BWG compared with the NC and maintained these parameters at levels comparable to the PC over the 0–14 d period. Although FCR increased numerically from 1.522 in the PC to 1.667 in the NC and was restored to 1.517 in the NC+phyG+XB200 treatment, the differences were not statistically significant. This lack of significance is likely due to the relatively low number of replicates, as the study was primarily designed to evaluate gut health parameters, which generally require fewer replicates than performance outcomes such as FCR.

## 5 Conclusions and implications

Feeding NSP-rich diets increased ileal digesta viscosity, intestinal permeability, impaired ileal mucosal morphology, and reduced weight gain in young broilers (0–14 d). Supplementation with phytase and a xylanase–β‑glucanase combination reduced ileal digesta viscosity and improved growth performance relative to NC. The response to xylanase–β‑glucanase followed a linear, dose-dependent pattern, with the higher inclusion level achieving performance comparable to the PC. The results suggest that xylanase and β-glucanase can be beneficial on top of phytase in NSP-rich diets to mitigate the negative effects of soluble NSP on gut health and growth performance in young birds.

## Disclosures

E. Vinyeta, Y. Dersjant-Li, A. Bello and K. Gibbs are employees of Danisco Animal Nutrition & Health (IFF), a global supplier of feed additive solutions.

## Statement for studies in humans/animals

The experiment was carried out in accordance with European Directive 2010/63/EU and the regulations in force in the Netherlands for the care and use of animals in research. In addition, the animal procedures and experimental protocols were evaluated and approved by the Ethical Committee on Animal Experiments (Ethische Toetsing Dierproeven) of Schothorst Feed Research B.V. prior to the commencement of research.

## CRediT authorship contribution statement

**Ester Vinyeta:** Writing – review & editing, Validation, Supervision, Resources, Methodology, Data curation, Conceptualization. **Yueming Dersjant-Li:** Writing – review & editing, Validation, Methodology, Formal analysis, Data curation, Conceptualization. **Abiodun Bello:** Writing – review & editing, Methodology, Data curation, Conceptualization. **Kirsty Gibbs:** Writing – review & editing, Resources, Methodology, Conceptualization. **Susan Arent:** Writing – review & editing, Methodology, Conceptualization. **Regiane R. Santos:** Writing – review & editing, Visualization, Validation, Supervision, Project administration, Methodology, Investigation, Formal analysis, Data curation, Conceptualization.

## Declaration of competing interest

The authors declare that they have no known competing financial interests or personal relationships that could have appeared to influence the work reported in this paper.
